# First‐in‐human application of double‐stranded RNA bacteriophage in the treatment of pulmonary *Pseudomonas aeruginosa* infection

**DOI:** 10.1111/1751-7915.14217

**Published:** 2023-01-13

**Authors:** Linlin Li, Qiu Zhong, Yunze Zhao, Juan Bao, Bing Liu, Zhuojun Zhong, Jing Wang, Lan Yang, Tingting Zhang, Mengjun Cheng, Nannan Wu, Tongyu Zhu, Shuai Le

**Affiliations:** ^1^ Shanghai Public Health Clinical Center, Shanghai Institute of Phage Fudan University Shanghai China; ^2^ Department of Clinical Laboratory, Daping Hospital Army Medical University Chongqing China; ^3^ CreatiPhage Biotechnology Co., Ltd Shanghai China; ^4^ Department of Laboratory Medicine The First Affiliated Hospital of Xi'an Jiaotong University Xi'an China; ^5^ Department of Microbiology Army Medical University Chongqing China; ^6^ Shanghai Medical College Fudan University Shanghai China

## Abstract

A double‐stranded RNA (dsRNA) phage phiYY is able to kill a pyomelanin‐producing *Pseudomonas aeruginosa* strain, which was isolated from a 40‐year‐old man with interstitial lung disease (ILD) and chronic lung infection. Phage therapy was used as a last resort for this patient. The three‐course nebulized phiYY treatment was used to reduce the bacterial burden and clinical symptoms of the patient. Recurrences of *P. aeruginosa* infections were observed 1–3 days post phage therapy. The recurrent isolates exhibited distinct antibiotic‐susceptibility profiles compared with the original strain yet were still susceptible to phiYY. This assay represents the application of dsRNA phage in the treatment of chronic lung infection, albeit the safety and efficacy of the dsRNA phage require further assessment.

## Graphical abstract

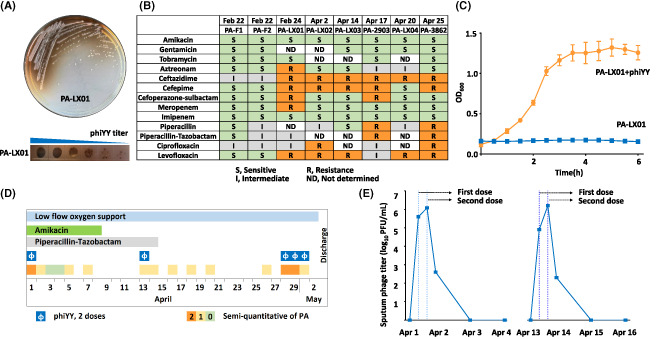

## INTRODUCTION

*Pseudomonas aeruginos*a is a key pathogen found in the respiratory tract of patients with chronic lung diseases (Smith et al., 2017). Chronic infections with *P. aeruginosa* are usually associated with bacterial biofilms, which results in increased antibiotic tolerance and the need of prolonged treatments (Rossi et al., 2021). Moreover, *P. aeruginosa* evolves during the respiratory chronic infection in individuals with cystic fibrosis (CF), and this adaptive evolution creates broad phenotypic and genomic changes that optimize the fitness of *P. aeruginosa* to inhabit in the CF airways (La Rosa et al., 2021; Pires et al., 2015). Approximately, 13% of chronically infected CF patients harbour pyomelanin‐producing *P. aeruginos*a during long‐term infection (Hocquet et al., 2016; Marvig, Dolce, et al., 2015; Marvig, Sommer, et al., 2015). These pyomelanin ‐producing strains lose between 200 kb and 600 kb genomic fragments, results in the accumulation of homogentisic acid (pyomelanin) and the loss of O‐antigen due to the loss of gene *hmgA* and *galU*, respectively (Shen et al., 2018). It has been reported that phage resistance due to the deletion of *galU* gene results in a lack of O‐antigen polysaccharide which is required for phage adsorption. The infections caused by these pyomelanin‐producing *P. aeruginos*a are difficult to cure due to airway damage, the loss of lung function in CF patients and the formation of biofilms.

## EXPERIMENTAL PROCEDURES

### Ethics approval statement

This study has been approved by the Ethics Committee of Shanghai Public Health Clinical Center (2020‐S157‐01), and the experimental procedures were completed under the prescribed protocol. Before receiving phage therapy, the patient has been communicated with and has agreed to sign an informed consent form after understanding the treatment plan.

### *P. aeruginosa* clinical isolates

Clinical strains of the patient during phage therapy were isolated from the patient's respiratory secretions, and the strains were isolated using MacConkey Agar plates, identified by mass spectrometry with Bruker MALDI (Matrix‐assisted laser desorption/ionization) Biotyper Rapid Microbial Identification System (Bruker Daltonik GmbH) and cultured in Luria‐Bertani medium at 37°C (Li et al., 2022; Yo et al., 2022). The antibiotic sensitivity test was performed by the agar disk diffusion method with reference to the Clinical and Laboratory Standards Institute (CLSI) standard.

The Semi‐Quantitative Clinical Cultures were used to measure the bacterial load as previously described (Serena et al., 2021). Briefly, culture plate was divided into four quadrants. Then the sputum was streaked from the first to the last quadrant using an inoculation loop. After incubation for 24 h, the number of quadrants with bacterial growth was assessed. A result of ‘0’ was recorded if no *P. aeruginosa* growth was observed on the plate; ‘1’ was reported if *P. aeruginosa* growth was only observed in the first quadrant; ‘2’ was reported if *P. aeruginosa* growth was observed on the first and second quadrants.

### Bacteriophage propagation and administration

The phage agent was prepared as previously described (Wu et al., 2021). Phage phiYY was cultured on the host strain PAO1r in the liquid LB medium, yielding lysates with titers of >1 × 10^9^ pfu/ml, which were purified with CIM® Anion exchange column QA (BIA Separations, Slovenia). Then, the concentrate was dialyzed with 0.9% sodium chloride physiological solution three times for 3 h each. Finally, the phage solution was filtered through a 0.22 μm filter and then aliquoted and packaged at the Good Manufacturing Practice (GMP) facility of Zhongshan Hospital of Fudan University, Shanghai, China. Phage phiYY was diluted in saline to 10 ml and administered via the vibrating‐mesh nebulizer.

### Determination of phage sensitivity on clinical strains

The susceptibilities of the target bacteria to phages were measured by the efficiency of plating (EOP) assay as previously described (Yang et al., 2020).

### Growth curve of isolated strain PA‐LX01

*Pseudomonas aeruginosa* strain PA‐LX01 was cultured to an OD600 of 0.1, then was infected with 200 μl of phiYY with a multiplicity of infection (MOI) of 0.01. Then the mixture was cultured at 37°C with shaking, and the OD600 of the bacterial solution at different time points was measured.

### Genome sequencing and analysis of *P. aeruginosa* clinical strains

The extraction of the bacterial genome was performed using a bacterial genomic DNA extraction kit (Sangon Biotech) and then sent to Shanghai Personalbio Technology for sequencing using the Illumina Miseq platform (∼1 Gbp/sample).

Then the genome similarity between the four strains (PA‐F1, PA‐F2, PA‐2903, PA‐3862) was estimated by Sourmash (Pierce et al., 2019). First, we computed sourmash signatures for every bacteria genome with two basic parameters (scaled = 1000, k‐mer = 31). Then, compare all the signatures to each other and plot a heatmap of the pairwise similarities between the genomes using Sourmash.

The sequence data is available in the NCBI under accession number JAPEVJ000000000, JAPFIE000000000, JAPFIF000000000 and JAPFID000000000 for PA‐2903, PA‐F1, PA‐F2, and PA‐3862, respectively.

### Determination of phage titre in the patient's sputum

Sputum was collected from patients at different time points. Then, the sputum was diluted to 5 ml with PBS, and centrifuged at 8000 × *g* for 2 min. Then, the phage titre was measured using EOP assay as described previously (Yang et al., 2020).

## RESULTS

A 40‐year‐old patient with progressive ILD, severe pulmonary *P. aeruginosa* infection, type I respiratory failure was referred for phage therapy as last resort. The patient was diagnosed with ILD 5 years ago, which progressed with aggravated pulmonary fibrosis and a concomitant *P. aeruginosa* infection after 4 years. The patient had received treatment with glucocorticoid (methylprednisolone) and multiple antibiotic regimes (amikacin, piperacillin‐tazobactam and sulfamethoxazole/trimethoprim) for 1 year in several local hospitals prior to the phage therapy at Shanghai Public Health Clinical Center.

**FIGURE 1:**
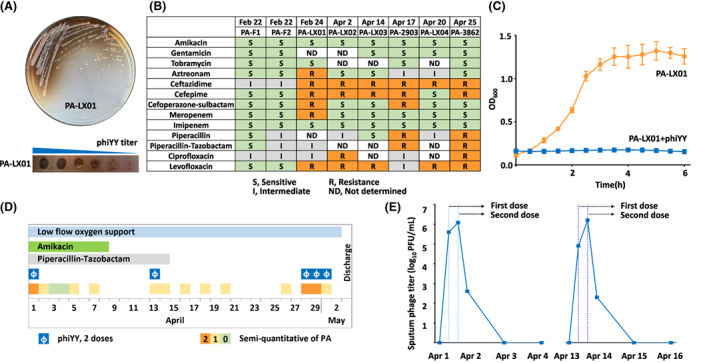
Characterization of the pyomelanin‐producing *P. aeruginos*a strain and the timeline of the dsRNA phage therapy. (A) The *P. aeruginos*a strain PA‐LX01 from the patient produces the brown pyomelanin, and phiYY lyses this strain efficiently. (B) The results of the antibiotic susceptibility testing for *P. aeruginos*a strains isolated before and after phage therapy. (C) Growth curve of PA‐LX01 strain in the presence or absence of phiYY. Each assay was repeated three times. (D) The timeline for the three courses of dsRNA phage therapy, which indicated the application of phage and antibiotics. (E) The titre of phage phiYY in the sputum before and after phage therapy.[Correction added on 17 January 2023, after first online publication: the labelling in figure 1C has been updated in this version.]

The *P. aeruginos*a strain PA‐LX01 isolated from the patient's sputum was identified as a pyomelanin‐producing, multidrug‐resistant pathogen (Figure 1A,B), which was resistant to all 100 dsDNA *Pseudomonas* phages in our library, but was susceptible to a dsRNA phage phiYY, which significantly inhibits the growth of PA‐LX01 in the liquid culture (Figure 1C). Thus, the lytic phage was propagated in cells growing exponentially in LB medium (Yang et al., 2016) and after 6 h, the phiYY phage was recovered as previously described (Wu et al., 2021).

Thus, this patient was treated with phiYY using a vibrating‐mesh nebulizer (Aerogen Solo). For the first course of treatment, phiYY was diluted in 10 ml 0.9% NaCl (Normal Saline) with a titre of 10^8^ PFU/ml, and was administered twice with an interval of 4 h. The patient experienced a transient fever (38.7°C) and returned to normal (36.5°C) within a few hours after indomethacin treatment. The phage treatment was followed by a transient elimination of *P. aeruginos*a in the sputum (Figure 1D) and a significantly improvement of the infection symptoms with decreased cough, expectoration and gasp.

Four days after the first phage therapy, the symptom of cough and expectoration recurred and pyomelanin‐producing *P. aeruginos*a was detected in the sputum. This isolate was still susceptible to phiYY, implying that phage resistance is not the primary cause of the bacterial recurrence. Thus, another course of phiYY nebulizer treatment, which was the same as the first one, was performed. The treatment course was once again associated with a transient fever, reduced bacterial burden, and further improved clinical symptoms. As a result, the antibiotic was completely stopped at April 14.

**FIGURE 2:**
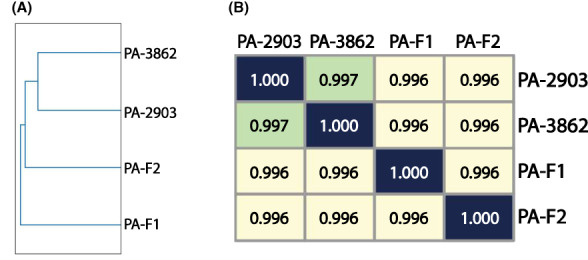
Comparative genomics of the pyomelanin‐producing *P. aeruginos*a strains indicates that their genomes are very similar. (A) The genome‐wide cladogram of four sequenced *P. aeruginos*a isolates based on the genome similarity calculated by sourmash. (B) The heatmap of the pairwise similarities between the genomes of the four sequenced *P. aeruginos*a isolates.

However, the infection was not eradicated and the recurrent strain PA‐LX03 was isolated and again shown sensitive to phiYY, while the antibiotic resistance profile was slightly lightened (Figure 1B). We sequenced four strains (PA‐F1, PA‐F2, PA‐2903, PA‐3862) selected during the phage therapy, and the four strains were very similar to each other with a similarity score of 0.996 ~ 0.997, but are not genetically identical according to the sourmash signatures (Figure 2). And their antibiotic susceptibilities are different (Figure 1B). Thus, the results are consistent with a previous report that the heterogenous populations in the chronic infected lungs had distinct phenotypes and genetic differences (Jorth et al., 2015), which might complicate the therapy. Moreover, the access of the phage to *P. aeruginos*a is not good enough given the thick biofilms that this bacterium forms in patient's lungs. Thus, the heterogenous populations and the formation of biofilm might both contribute to the persistent infection.

We examined the presence of phiYY in the sputum, found that the phage load decreased by ~10,000‐fold within 1 day after the treatment and could not be detected after 48 h (Figure 1E). Therefore, after the agreement of the patient, we prolonged phage therapy to 3‐day with 2 doses of phiYY daily. After 3 days of treatment, the patient was discharged with relieved symptoms of infection. A half year later, he received lung transplantation and recovered well without any lung infection during the follow‐up.

## DISCUSSION

Phage therapy is a promising approach to treat bacterial infections (Uyttebroek et al., 2022). To be noted, all reported phage therapies were performed with DNA phages, as DNA phages are the most abundant and diverse group of phages (Dion et al., 2020). However, phiYY is currently the only dsRNA phage that infects the O‐antigen deficient *P. aeruginosa* among the eight dsRNA phages (Yang et al., 2016, 2020). Considering the large population of CF and chronic lung infection patients, who might be infected by the pyomelanin‐producing *P. aeruginos*a (Marvig, Dolce, et al., 2015; Marvig, Sommer, et al., 2015), phiYY could be an appropriate agent to treat these patients.

The improved clinical symptoms, discontinuations of antibiotics and reduced bacterial burden all indicated that phiYY was effective in killing the bacteria. However, the fever occurred right after each treatment is likely due to the instantly bacterial lysate released by phage progeny production, or immune response caused by dsRNA phage itself (Kimura‐Takeuchi et al., 1992). However, the molecular mechanism behind the observed response requires further verification in animal models.

There are some reasons for the failure to completely eradicate the *P. aeruginos*a. Since all the isolates were sensitive to phiYY, phage resistance might not be the primary cause. The genomes of four strains are different (Figure 2), which indicates that the heterogenous populations might contribute to the persistent infection.

Secondly, the duration of phage therapy for lung infections is important (Rao et al., 2022; Wu et al., 2021). For acute infection, 1‐day phage therapy would be effective (Wu et al., 2021), while for the chronic infections, prolonged course is needed (Dedrick et al., 2019). In this case, we only applied 1‐day or 3‐day phage therapy, which were effective in controlling the symptoms for a short period yet unable to eliminate the infection. Thus, prolonged phage therapy is needed to treat the chronic bacterial infection (Dedrick et al., 2019; Suh et al., 2022).

## AUTHOR CONTRIBUTIONS

**Linlin Li:** Data curation (equal); investigation (equal); methodology (equal); visualization (equal); writing – original draft (equal). **Zhong Qiu:** Data curation (equal); investigation (equal); methodology (equal); visualization (equal); writing – original draft (equal). **Yunze Zhao:** Data curation (equal); investigation (equal); methodology (equal); visualization (equal); writing – original draft (equal). **Juan Bao:** Data curation (equal); investigation (equal); methodology (equal); visualization (equal); writing – original draft (equal). **Bing Liu:** Investigation (supporting); methodology (supporting); resources (supporting). **zhuojun zhong:** Investigation (supporting); methodology (supporting); resources (supporting). **Jing Wang:** Investigation (supporting); methodology (supporting); resources (supporting). **Lan Yang:** Investigation (supporting); methodology (supporting); resources (supporting). **tingting Zhang:** Investigation (supporting); methodology (supporting); resources (supporting). **mengjun Cheng:** Investigation (supporting); methodology (supporting); resources (supporting). **nannan Wu:** Conceptualization (equal); funding acquisition (equal); writing – original draft (equal). **tongyu Zhu:** Conceptualization (equal); funding acquisition (equal); writing – original draft (equal). **Shuai Le:** Conceptualization (equal); funding acquisition (equal); writing – original draft (equal).

## CONFLICT OF INTEREST

No potential conflict of interest was reported by the authors.
